# Case Report: Repeated imaging and surgical treatment of a vascular hamartoma arising from the cervical vertebral column of a cat

**DOI:** 10.3389/fvets.2026.1940123

**Published:** 2026-09-09

**Authors:** Annika Katharina Schmitz, Gesine Buhmann, Andrea Trapp, Kaspar Matiasek, Konrad Jurina

**Affiliations:** 1 Centre for Clinical Veterinary Medicine, Neurology Unit, Ludwig-Maximilians-Universität München, Munich, Germany; 2 AniCura Tierklinik Haar GmbH, Neurology Department, Haar, Germany; 3 Section of Clinical & Comparative Neuropathology, Centre for Clinical Veterinary Medicine, Ludwig-Maximilians-Universität München, Munich, Germany

**Keywords:** cervical myelopathy, feline, hamartoma, surgical decompression, vascular malformation, vertebral angiomatosis

## Abstract

A 3-year-old male neutered Domestic Shorthair cat was presented with a 1-year history of progressive right-lateralized proprioceptive ataxia, ambulatory tetraparesis and cervical spinal pain, with sudden deterioration shortly before presentation. Neurological examination was consistent with a right-sided C6–T2 myelopathy. Magnetic resonance imaging (MRI) revealed a right-sided extradural myelocompressive lesion at the level of the C5 vertebra, associated with osseous proliferation arising from the right lamina and vertebral body, causing severe spinal cord compression and leftward deviation of the spinal cord. The mass was surgically resected, and histopathology confirmed a vertebral vascular hamartoma. Clinical signs improved after a brief period of postoperative worsening. One year later, neurological deficits recurred. MRI and computed tomography (CT) confirmed regrowth of the vertebral lesion. A second surgical excision was performed, and histopathology again confirmed a vertebral vascular hamartoma. To the authors' knowledge, this is the first report describing follow-up imaging and repeated surgical intervention, with histopathological confirmation at both procedures, in a cat with recurrent cervical vertebral vascular hamartoma. It highlights vertebral vascular hamartoma as an important differential diagnosis for young cats with progressive myelopathy, extradural spinal cord compression and vertebral osseous proliferation, and suggests that surgical intervention can result in meaningful clinical improvement despite possible regrowth after incomplete resection.

## 1 Introduction

Hamartomas are generally considered developmental tumor-like malformations characterized by a benign, disorganized overgrowth of mature tissue elements native to the affected organ. Vascular hamartomas are composed of mature but structurally abnormal vascular tissue, which may form a proliferative, space-occupying lesion and, when involving bone, may exhibit focal osteolytic changes (1–4). These lesions are generally slow-growing and may remain subclinical for extended periods, with clinical manifestation largely influenced by their location and size (5). Because vascular tissue is widely distributed throughout the body, vascular hamartomas may occur in a wide range of anatomical locations (6–19) including the central nervous system (20–25) and vertebral column (3, 26–28). Vertebral vascular hamartomas are rare, particularly in cats, and may lead to neurological deficits when associated with spinal cord compression (3, 26, 28). A presumptive diagnosis of vertebral vascular hamartoma or angiomatosis is primarily based on advanced imaging, particularly magnetic resonance imaging (MRI) and computed tomography (CT). However, imaging findings are not pathognomonic and may resemble other inflammatory, traumatic, or neoplastic spinal lesions. Histopathological examination of surgically obtained tissue is required for definitive diagnosis (3, 26, 28–30). Morbidity associated with vascular hamartomas is primarily driven by their local mass effect and potential infiltration of adjacent tissues (2, 22, 23). Surgical resection is considered the treatment of choice for symptomatic lesions or when diagnostic uncertainty is present. Complete excision is associated with a good outcome and low risk of recurrence (9, 13, 17, 31–33), whereas incomplete resection may result in regrowth (34, 35).

Due to their uncommon occurrence and potential for recurrence, detailed clinical characterization and long-term follow-up are important. To the best of the authors' knowledge, this is the first report describing follow-up imaging and repeated surgical intervention, including histopathology, for a relapsing vertebral vascular hamartoma in the cervical vertebral column of a cat.

## 2 Case description

### 2.1 History and neurological examination

A 3-year-old male neutered Domestic Shorthair cat was presented with a history of progressive ataxia and spinal pain. The owners reported that the cat had fallen from a scratching post approximately one year prior to presentation, after which an ataxic gait and pain on cervical spinal palpation were noted. Medical management following the traumatic event included non-steroidal anti-inflammatory drugs and monthly administration of frunevetmab (Solensia), resulting in partial improvement; however, ataxia and pain persisted. During the two weeks preceding presentation, clinical signs progressively worsened. On the day of admission, the cat exhibited acute deterioration characterized by marked pain with vocalization and severely reduced ambulatory ability.

General physical examination at time of presentation was unremarkable. Neurological examination revealed normal mentation, with spontaneous knuckling of the right thoracic limb. Gait assessment showed ambulatory tetraparesis more pronounced on the right side, proprioceptive ataxia with knuckling of the right forelimb and a tendency to fall to the right. Postural reactions were absent in the right thoracic limb and reduced in the remaining limbs. Segmental spinal reflexes were absent in the right thoracic limb and normal in the remaining limbs. Neuroanatomical lesion localization was consistent with a right-lateralized lesion affecting the C6–T2 spinal cord segments.

At this time, a complete blood cell count, serum biochemistry profile, screening for feline immunodeficiency virus and feline leukemia virus, and thoracic radiographs were performed. Apart from mild hyperproteinemia with increased globulin concentration and mild hemoconcentration, tests were unremarkable.

## 3 Diagnostic assessment

### 3.1 Diagnostic imaging

**Figure 1:**
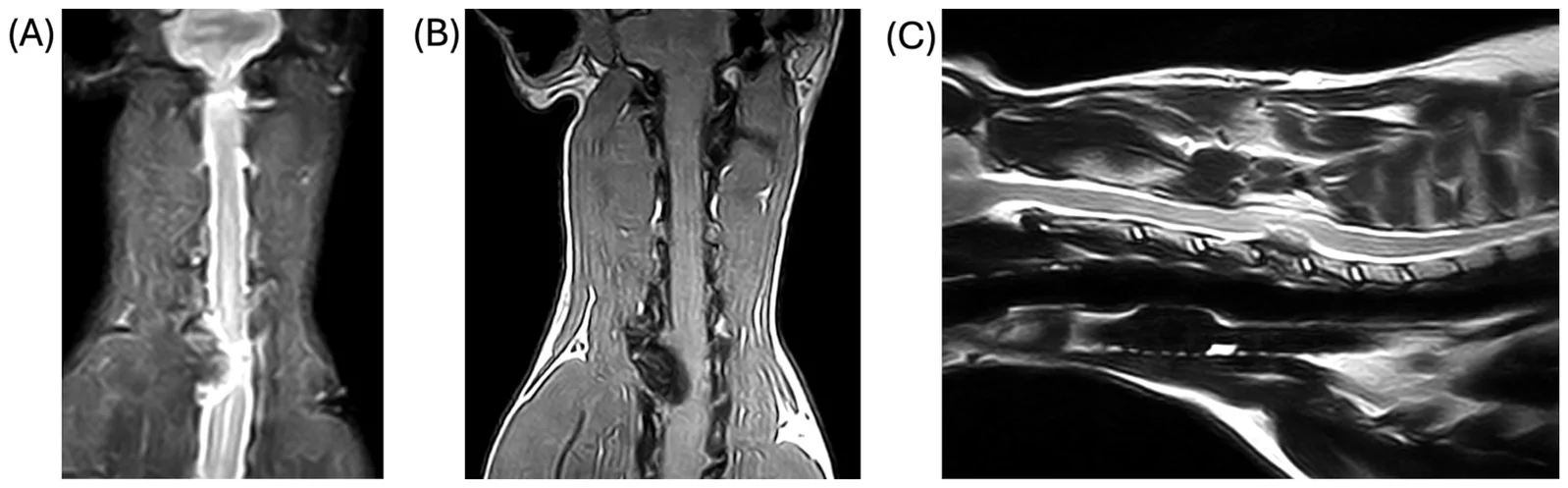
Initial MRI of the cervical and cranial thoracic vertebral column. Dorsal T2- **(A)**, dorsal 3D-HYCE- **(B)** and sagittal T2-weighted **(C)** MRI of the cervical vertebral column. Images demonstrate a right-sided extradural lesion associated with the C5 vertebra, resulting in marked right-lateralized spinal cord compression and leftward displacement of the spinal cord. The lesion is hypointense compared to the spinal cord on T2-weighted and 3D-HYCE-weighted images.

**Figure 2:**
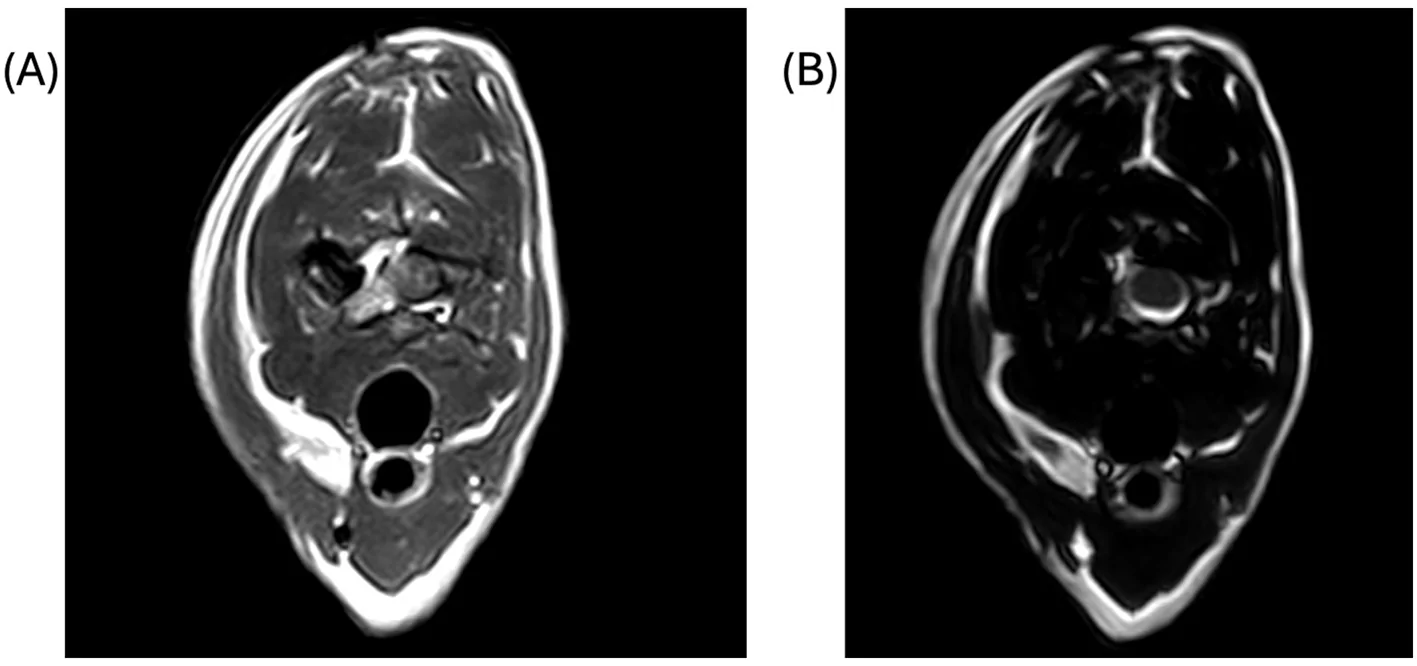
Transverse MRI of the cervical vertebral column at the level of C5. Transverse post-contrast T1-weighted image **(A)** demonstrates marked right-sided extradural spinal cord compression caused by a vertebral lesion arising from the right lamina and vertebral body, with leftward displacement of the spinal cord. The lesion is hypointense on the T1-weighted image. Thickening and contrast enhancement of the right C5 spinal nerve root are evident, whereas the osseous lesion itself shows no obvious contrast enhancement. Transverse T2-weighted image **(B)** demonstrates ventral accumulation of cerebrospinal fluid adjacent to the site of compression.

Low-field MRI (0.25 Tesla; Vet-MR Grande, Esaote) of the cervical, cranial thoracic vertebral column and cranium in prone position was performed in sagittal, transverse, and dorsal views in FSE T2-, FAST STIR-, 3D HYCE-, SE T1-, and 3D SST1-weighted sequences. The T1-weighted sequences were obtained before and after intravenous gadolinium (Dotarem) administration (0.2 mmol/kg i.v.). At the level of the C5 vertebra, an osseous proliferation arising from the right lamina and vertebral body was identified, resulting in marked right-lateralized extradural compression and deviation of the spinal cord to the left. The mass was hypointense to the spinal cord on STIR-, T1- and T2-weighted images (Figure 1). Additional findings were a thickening of the right C5 and C6 spinal nerve roots. An accumulation of cerebrospinal fluid (CSF) was detected ventrally, extending cranially and caudally from the site of compression, with complete effacement of CSF and epidural fat signal at the level of the mass (Figure 2). These findings were best appreciated on the 3D HYCE sequence. Contrast enhancement was observed in thickened C5 and C6 nerve roots as well as in the meninges along the entire length of the compression site. The mass lesion itself showed no contrast uptake. Overall, findings were consistent with a right-sided extradural mass lesion originating from the C5 vertebra. Differential diagnoses included primary vertebral neoplasia, such as osteosarcoma, chondrosarcoma or fibrosarcoma, lymphoma, vertebral vascular hamartoma, inflammatory or infectious granulomatous disease, and reactive post-traumatic osseous proliferation.

### 3.2 Surgical procedure and perioperative management

Surgery was performed under general anesthesia induced with midazolam and propofol i.v. and maintained with inhalational anesthesia with isoflurane under mechanical ventilation. Preoperative analgesia consisted of methadone (0.2 mg/kg i.v.), and perioperative analgesia was provided by a ketamine constant rate infusion (5 μg/kg/min).

The cat was positioned in sternal recumbency, and a dorsal approach to the cervical vertebral column was performed. The spinous process of C5 was identified fluoroscopically. The laminae from C4 to C6 were exposed, and a modified right-sided dorsal laminectomy of C5 was performed using a high-speed burr (ELAN 4, Aesculap AG, Tuttlingen, Germany). The bone in the affected region appeared markedly spongious, with a firm osseous component underlying the spinal cord. Representative tissue samples were collected for neuropathological examination using a Kerrison rongeur (Aesculap AG, Tuttlingen, Germany).

**Figure 3:**
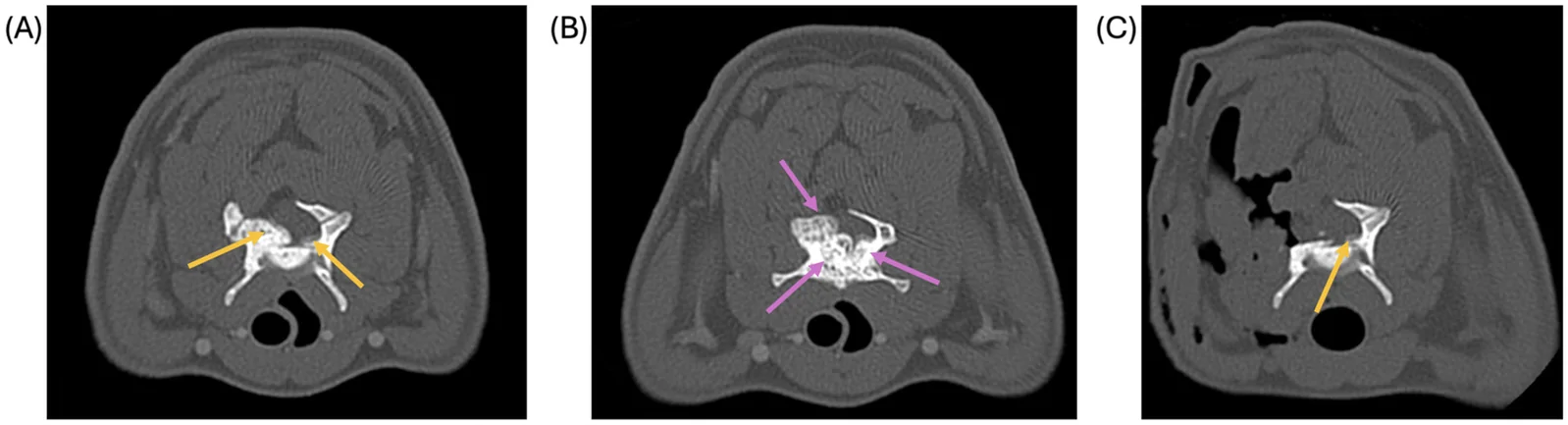
CT of the cervical vertebral column after first surgery and before and after repeated surgery at the level of C5. Transverse CT images in bone window at the level of C5. Image **(A)**, obtained after initial surgery, shows residual irregular osseous proliferation (orange arrows) associated with the C5 vertebra. Follow-up CT before the second surgery **(B)** demonstrates marked irregular, cloud-like to sponge-like osseous proliferation involving the right vertebral arch, lamina, pedicle and vertebral body (purple arrows), resulting in severe stenosis of the vertebral canal. Postoperative CT after the second surgery **(C)** shows improved vertebral canal dimensions following decompression, with a small amount of residual ventral osseous proliferation remaining within the vertebral canal (orange arrow).

Following biopsy collection, the spinal cord remained markedly compressed by abnormal osseous tissue. Further decompression was therefore performed using a high-speed burr and Kerrison rongeur. Bone was removed until the spinal cord and the affected nerve root were visually free of direct compression and adequate space was confirmed by gentle probing. Moderate hemorrhage from tissue surrounding the C5 spinal nerve root and adjacent cancellous bone was controlled with hemostatic measures, including bone wax. Complete removal of the ventrally located abnormal tissue was not considered safely achievable. An autologous fat graft harvested from the cervical subcutaneous tissue through the same surgical incision was placed over the exposed dura and laminectomy defect, followed by lavage and routine layered closure. Postoperative CT confirmed satisfactory decompression, although a small amount of residual irregular osseous proliferation associated with the C5 vertebra remained (Figure 3A). Postoperatively, the cat received methadone (0.2 mg/kg i.v. QID), prednisolone (0.5 mg/kg p.o. SID) and pregabalin (2 mg/kg p.o. TID). The ketamine (5 μg/kg/min i.v.) constant rate infusion was continued for 12 h. Bladder function was monitored during hospitalization.

Following surgery, the cat initially showed neurological deterioration, becoming laterally recumbent with tetraplegia, flaccid thoracic limbs and reduced thoracic limb segmental reflexes, pelvic limb spasticity with normal segmental reflexes, and inability to lift its head. Voluntary urination and defecation were initially absent. The urinary bladder was managed by manual expression, and manual rectal evacuation was required during the early recovery period. Intensive supportive care and physiotherapy were initiated immediately. On the day of discharge, four days postoperatively, the cat showed mild neurological improvement, with limited voluntary head movement. The thoracic limbs, which had initially been flaccid, had developed spasticity and the cat remained tetraplegic. Therapy was continued by the owners and included at-home care and intensive physiotherapy.

Over the following weeks, gradual neurological improvement was observed. 11 days postoperatively, the cat remained tetraplegic but was able to maintain sternal recumbency with active head movement. The spasticity of all four limbs was still present but was improving, as indicated by an increased passive range of motion during physiotherapy. By approximately three weeks postoperatively, voluntary motor function had returned, and the cat was non-ambulatory tetraparetic. Voluntary urination and defecation had also resumed. At six weeks postoperatively, the cat had regained independent tetraparetic ambulation, although the gait remained crouched with occasional falling to the right, and proprioceptive positioning was still absent.

Approximately eight weeks postoperatively, the cat had regained a normal posture during ambulation. Proprioceptive positioning of the right thoracic and pelvic limbs remained mildly delayed, and subtle intermittent dragging of the right pelvic limb persisted thereafter.

### 3.3 Histopathology

**Figure 4:**
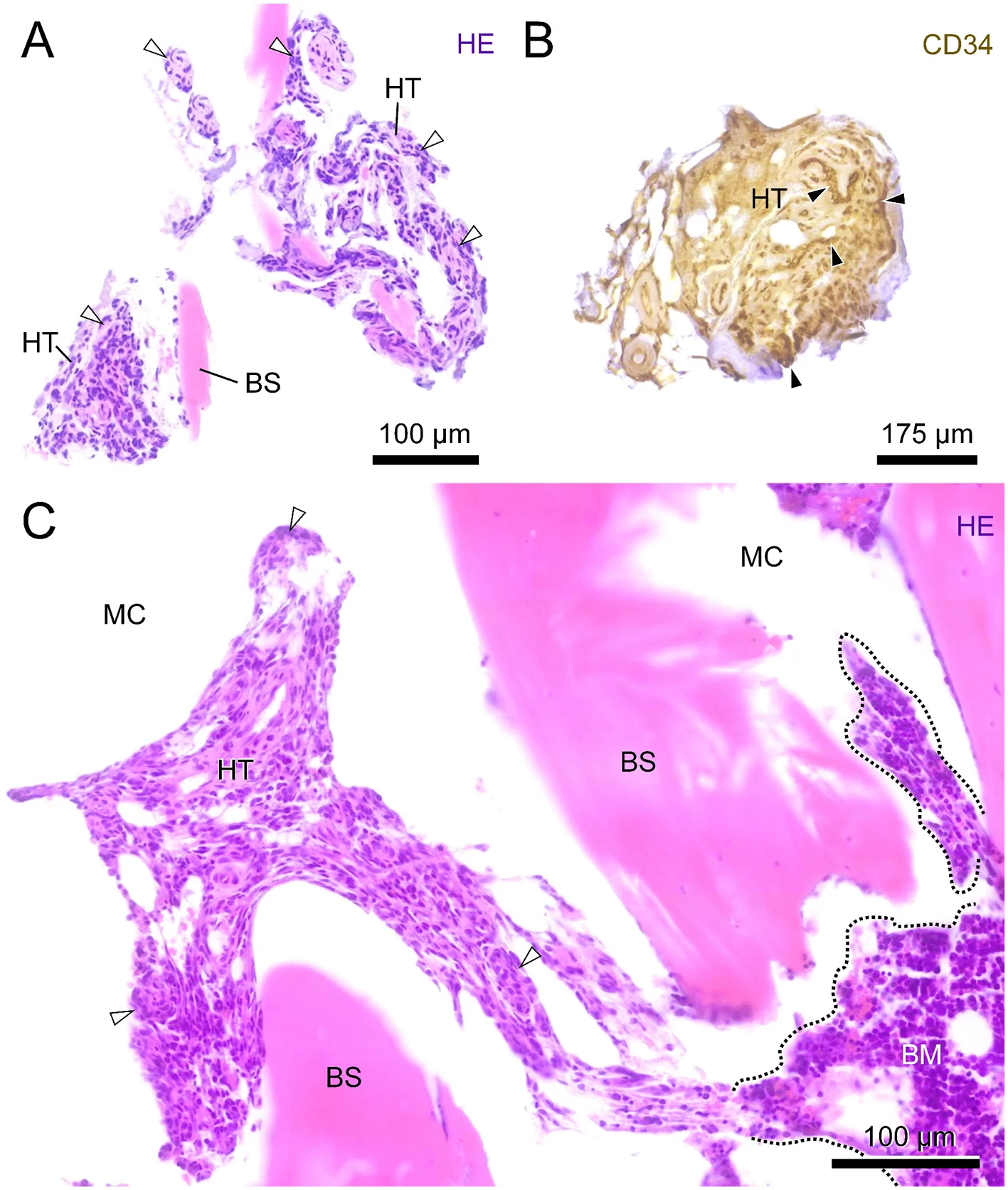
Biopsy findings on first **(A, B)** and second surgery **(C)**. In general, the hamartomatous tissue (A-C: HT) consists of branching bundles of irregular and often non-luminal proliferates of mostly arteriolar blood vessels (A, C: white arrowheads) that flag-up on immunohistochemistry for the early vessel marker CD34 (B: black arrowheads). Intralesional bone spicules (A: BS) often appear disproportionately thin, while those of the outer cortex (C: BS) are remarkably thickened. In some fragments, hamartomatous proliferates (C: HT) mingle with hematopoietic bone marrow (C: BM) within enlarged medullary cavities (C: MC). Stains: A,C: Hematoxylin-eosin (HE); B: Immunohistochemistry for CD34 using a horseradish peroxidase- diaminobenzidine detection kit (brown).

Resected tissue samples were immersed in formalin and submitted for histopathological evaluation. On arrival at the laboratory, the samples were trimmed and subjected to automatic tissue processing for paraffin embedding, obtainment of 3 μm thick sections and routine histopathological staining for bone and bone marrow lesions (hematoxylin eosin, Giemsa stain) (36), followed by immunohistochemical staining for the transmembrane glycoprotein CD34. Microscopic examination revealed multiple fragments of cancellous bone with enlarged medullary cavities containing an irregular, branching proliferation of focally non-luminal capillary and arteriolar blood vessels embedded in a loose and occasionally myxoid mesenchymal stroma. Intralesionally trapped bone spicules appeared comparatively thin and often scalloped. In the outer parts of the fragments, focal hyperostotic lesions were seen. Throughout all areas, the blood vessels stained positive for CD34 (37). These findings were consistent with the diagnosis of a vertebral vascular hamartoma with hyperostotic cortical remodeling (Figure 4A, B), comparable to previously reported feline vertebral vascular lesions (3, 29). However, histopathological overlap between vascular hamartomas and vascular malformations is recognized, and definitive distinguishing criteria have not been established (4).

### 3.4 Recurrence, repeated diagnostic imaging and second surgery

At 12 months after the initial surgery, the cat developed recurrent neurological deficits, consistent with suspected lesion regrowth. The owner reported a one-week history of progressive gait abnormalities with proprioceptive ataxia and dragging of the right thoracic and pelvic limbs following a minor fall; however, prior to this event, the cat had shown marked clinical improvement, with only subtle residual intermittent dragging of the right pelvic limb. No medical treatment was being administered at this time. Neurological examination revealed normal mentation and behavior. Postural abnormalities included spontaneous knuckling of the right thoracic limb and a tendency to fall to the right. Gait assessment demonstrated a moderately ambulatory right-sided hemiparesis. Segmental spinal reflexes were reduced in the right thoracic limb. Neuroanatomical localization was consistent with a right-lateralized lesion affecting the C6–T2 spinal cord segments, as at the initial presentation.

Repeated MRI of the cervical vertebral column was performed using the same imaging protocol as initially. Imaging findings were largely unchanged compared with the initial MRI examination and were consistent with recurrent marked extradural compression at C5 due to regrowth of the previously diagnosed vertebral vascular hamartoma. The recurrent lesion arose from the right lamina and vertebral body and resulted in marked right-lateralized extradural spinal cord compression with leftward deviation of the spinal cord. The mass remained hypointense to the spinal cord on STIR-, T1- and T2-weighted images and showed no obvious contrast uptake. Persistent thickening and contrast enhancement of the right C5 and C6 spinal nerve roots were again observed.

Following MRI, CT of the cranial cervical vertebral column was performed in sternal recumbency using a 16-slice CT scanner (Optima 520, GE Hangwei Medical Systems Co., Ltd., Beijing, China). CT confirmed a marked irregular, cloud-like to sponge-like osseous proliferation associated with the C5 vertebra, involving the right dorsal and lateral vertebral arch, lamina, pedicle and vertebral body. The lesion resulted in marked secondary stenosis of the vertebral canal and extended around most of the canal, with relative sparing of the left dorsolateral vertebral arch. The vertebral canal diameter was reduced from 11.7 mm at the widest unaffected level to 6.8 mm at the most stenotic point. The inner osseous margin was markedly irregular, and the intervening bone showed reduced attenuation. No extraskeletal soft tissue component was evident (Figure 3B).

A second surgical procedure was performed under general anesthesia with perioperative analgesic management similar to that used during the initial intervention. Because the ventral and right-sided components of the lesion had not been completely accessible during the first procedure, a modified lateral approach to the cervical vertebral column was selected for the second surgery, as previously described (38). Marked adhesions consisting of fibrous and adipose tissue were present at the previous laminectomy site. A firm osseous proliferation was identified and partially resected using a high-speed burr and Kerrison rongeur, and tissue samples were submitted for histopathological examination. An extended hemilaminectomy from the caudal aspect of the C4 vertebra to the cranial aspect of the C6 vertebra was performed until the spinal cord and nerve root were visually decompressed. Moderate venous hemorrhage associated with the spinal nerve roots was controlled with local hemostatic agents. A fat graft was placed covering the spinal cord and laminectomy defect, and routine closure was performed.

Histopathology of the second biopsy confirmed recurrence of the vertebral vascular hamartoma (Figure 4C).

Postoperative CT confirmed satisfactory decompression with improved vertebral canal dimensions; however, a small amount of residual osseous proliferation arising from the vertebral body persisted ventrally within the vertebral canal (Figure 3C).

Recovery after the second surgery was markedly faster than after the initial procedure, with no evidence of postoperative neurological deterioration. At re-examination three weeks after surgery, the cat had shown marked clinical improvement, with only mild proprioceptive ataxia and mildly delayed proprioceptive positioning of the right thoracic and pelvic limbs.

Physiotherapy was continued, and at the most recent follow-up, five months postoperatively, the cat remained clinically stable with only mild residual proprioceptive ataxia. At the time of manuscript preparation, no longer-term follow-up was available because only five months had elapsed since the second procedure. Given the presence of residual abnormal tissue on postoperative CT, adjunctive radiation therapy (RT) was discussed with the owners. However, this option was declined, and clinical and imaging follow-up was elected instead.

## 4 Discussion

Vascular hamartomas are generally considered developmental, non-neoplastic malformations that may become clinically apparent when progressive enlargement causes compression of adjacent structures (39). In the present case, the cervical vertebral location resulted in marked extradural compressive myelopathy.

Advanced imaging was essential to define the vertebral origin of the lesion and the extent of extradural spinal cord compression. MRI provided detailed information on spinal cord displacement, enlargement of the adjacent nerve roots and contrast enhancement of the extradural tissues, whereas CT was particularly useful for assessing the osseous component of the lesion. Despite its vascular origin, the lesion showed a predominantly osseous appearance, which may reflect secondary remodeling of the vertebral bone in response to the intraosseous vascular proliferation (40, 41). Previous feline cases of vertebral angiomatosis similarly demonstrated sponge-like osseous proliferation associated histologically with benign vascular proliferation, bone fibrosis and new bone formation (29).

In the present case, this interpretation is supported by histopathological identification of irregular intramedullary microvessels within enlarged marrow spaces, accompanied by thin bony trabeculae with focal nodular thickening and surrounding hyperostotic bone, indicating that the imaging appearance reflected a combination of abnormal vascular tissue and associated osseous remodeling. Moreover, marked thickening and contrast enhancement of the right C5 and C6 spinal nerve roots were present both initially and at recurrence. In cats, nerve root enlargement and enhancement have previously been reported in association with neoplastic or inflammatory diseases and may therefore mimic peripheral nerve sheath tumor, lymphoma or neuritis (42–46). However, these findings are not pathognomonic. Reversible nerve root and peripheral nerve enlargement with contrast enhancement has been described in a cat secondary to mechanical nerve root compression, with histopathology confirming interstitial nerve edema and follow-up MRI showing complete resolution after decompression (42). Therefore, the nerve root changes in the present case may have represented secondary edema and/or periradicular inflammation caused by chronic compression and altered blood-nerve barrier permeability rather than a separate primary nerve root disease.

In addition to secondary edema, alternative pathophysiological mechanisms for the persistent nerve root enlargement and enhancement include localized venous congestion resulting from mechanical venous outflow obstruction (47), reactive dural or periradicular fibrosis, or direct microvascular proliferation of the hamartoma extending along the foraminal tissues (48).

Furthermore, the lack of prominent contrast enhancement within the vertebral lesion itself represents an intriguing imaging observation. While unexpected for a hypervascular lesion, minimal, irregular, or delayed enhancement patterns are well-documented in atypical or slow-flow vascular anomalies (49). In our patient, this lack of prominent enhancement may be attributed to slow-flow hemodynamics within the capillary or venous channels or the high proportion of dense osteosclerotic remodeled bone and thick fibrous stroma relative to active vascular lumens, which can visually mask contrast perfusion (50). Taken together, these imaging features are not specific for vertebral vascular hamartoma. Therefore, histopathological examination remained necessary to establish the definitive diagnosis (3, 26, 28, 30).

For symptomatic vascular hamartomas or lesions causing mass effect, surgical excision is generally considered the treatment of choice, both to achieve decompression and to obtain tissue for definitive histopathological diagnosis. Complete resection has been associated with favorable outcomes, whereas incomplete resection may predispose to regrowth and the need for further intervention (34, 35). Vascular hamartomas of the vertebral column appear to follow a similar outcome pattern. This is illustrated by two previously reported feline cases of vertebral vascular hamartoma. In one case, complete excision of a cervical lesion was curative, with a 2-year follow-up period without recurrence of clinical signs, whereas in another case involving the thoracic vertebral column, relapse occurred seven months after surgery and was successfully managed with a second surgical intervention (3, 26). In the present case of a vertebral vascular hamartoma affecting the cervical vertebral column, recurrence of clinical signs was observed approximately 12 months after the initial surgery. Diagnostic imaging confirmed suspicion of lesion regrowth, and a second surgical intervention was performed. Histopathological examination of both specimens was consistent with a vertebral vascular hamartoma. Follow-up CT after the second procedure revealed residual lesion at the level of the C5 vertebral body, indicating incomplete resection. The choice of surgical approach may also have influenced the extent of resection. At the initial procedure, a modified right-sided dorsal approach was selected, but the ventral and ventrolateral components of the lesion remained difficult to access safely and residual osseous tissue was documented on postoperative CT. In retrospect, preoperative CT may have provided a more complete assessment of the circumferential osseous involvement of C5 and may have influenced surgical planning. A lateral approach may have facilitated access to the deeper ventrolateral component of the lesion and potentially allowed more extensive resection. This consideration contributed to the decision to use a modified lateral approach at the second surgery. However, because abnormal tissue also arose from the vertebral body and extended ventrally within the vertebral canal, complete excision may still have carried an unacceptable risk of injury to the spinal cord, nerve roots or supporting vertebral structures. Recurrence therefore likely reflected both persistence of residual malformative tissue and limitations imposed by surgical accessibility. These observations highlight the potential value of postoperative monitoring when complete excision cannot be confirmed. Given the limited number of reported cases, no evidence-based follow-up interval can be recommended. This case suggests that follow-up imaging may be valuable when residual tissue is suspected. In human medicine, vascular imaging may be used in addition to MRI to characterize the angioarchitecture of spinal vascular malformations and guide treatment planning (51). Although angiographic imaging was not performed in the present case and is rarely used in clinical veterinary neurology, repeated MRI and CT were valuable for documenting residual tissue and subsequent lesion regrowth. The marked neurological deterioration observed after the first surgery constituted a significant postoperative complication. While objective data regarding early postoperative deterioration and spinal cord reperfusion injury in feline patients are extremely sparse, valuable clinical parallels can be drawn from canine neurosurgery, where up to 54% of dogs undergoing cervical decompressive surgery experience a transient decline in neurological status within 48 h postoperatively (52). Potential underlying mechanisms in domestic animals include intraoperative spinal cord manipulation, postoperative spinal cord edema or mechanical expansion, transient spinal hypoperfusion or vascular compromise, and postoperative vertebral instability (52). Decompression of chronically compressed, ischemic spinal cord tissue can also precipitate a reperfusion-associated spinal cord injury (52–55). In human medicine, an extreme manifestation of this cascade is termed “white cord syndrome”, which is characterized by acute postoperative paralysis and hyperintense intramedullary T2-weighted MRI signals. This syndrome is attributed to an abrupt restoration of blood flow to chronically ischemic tissue, leading to blood–spinal cord barrier disruption, localized edema, and secondary oxidative and inflammatory injury. However, because an immediate postoperative MRI was not performed in the present case to rule out other compressive pathologies or document intramedullary T2-hyperintensities, the exact postoperative mechanism remains speculative.

In human medicine, vascular anomalies are broadly classified into vascular tumors and vascular malformations according to biological behavior and endothelial proliferation. Although this classification cannot be directly applied to veterinary vertebral vascular hamartomas, it supports the interpretation of such lesions as non-neoplastic malformative processes (56–58). This distinction is relevant for the present case, as the recurrent clinical signs were most likely attributable to regrowth of residual malformative tissue rather than malignant transformation. Nevertheless, the terminology and classification of vascular proliferative lesions remain challenging in veterinary neuropathology.

Histopathologically, while diffuse CD34 immunoreactivity confirms the lesion's endothelial origin, the absence of cytological atypia or mitotic activity and the presence of mature capillary- and arteriolar-type vessels embedded in abundant intervening stroma favor a non-neoplastic developmental vascular anomaly (37, 57) over a true benign neoplasm like cavernous hemangioma, where vessel walls typically directly abut with minimal to no intervening parenchyma (4). While these features historically supported the diagnosis of “vascular hamartoma”, they do not reliably distinguish it from a localized vascular malformation. Nonetheless, the ongoing evolution and debate regarding this nomenclature must be acknowledged. Indeed, a recent review questioned the term “vascular hamartoma” in veterinary neuropathology because of its substantial histopathological overlap with localized vascular malformations and the lack of clearly distinguishing histopathological criteria (4). Marr et al. (4) categorized proliferative vascular disorders of the central nervous system as reactive vascular proliferations, vascular malformations including benign neoplasms and hamartomas, and neoplastic disorders. Under the current ISSVA framework, this lesion in the present case most closely aligns with a non-neoplastic, slow-flow vascular malformation (59). Nevertheless, we retained the term “vertebral vascular hamartoma” to maintain consistency with previous feline vertebral literature (3, 28), while acknowledging that biologically the lesion may be regarded as part of the broader spectrum of non-neoplastic vascular malformations (4, 50).

In the present case, adjunctive RT was discussed with the owners because residual abnormal tissue remained after the second surgery; however, this option was declined. Veterinary evidence regarding RT for vertebral vascular lesions in cats remains extremely limited. A single feline case of vertebral angiomatosis (classified as a vascular malformation) (4) was treated with surgical decompression followed by adjuvant megavoltage RT (48 Gy in 16 fractions), with an excellent outcome and no reported recurrence during more than 26 months of follow-up (60). In addition, successful interstitial brachytherapy has been described for a recurrent dermal vascular hamartoma in a horse (61). In human medicine, RT is considered an acceptable treatment option for aggressive vertebral hemangiomas (62), a term often used synonymously with vascular malformations in human spinal pathology due to their arbitrary and overlapping morphological distinction (4). This intervention is particularly indicated in cases with gradual neurological deterioration, incomplete resection or when surgery is not feasible (62). However, neurological recovery after RT may be delayed, and whether these findings can be extrapolated to feline vertebral vascular malformations remains unknown (62). Although the favourable outcome reported in the feline case is noteworthy, the available veterinary evidence is insufficient to support general recommendations regarding the efficacy, optimal dose, indications, or long-term safety of RT for feline vertebral vascular malformations.

In conclusion, vertebral vascular hamartoma should be considered as a rare differential diagnosis in young cats with progressive cervical myelopathy and a vertebral extradural compressive lesion with osseous involvement. Surgical decompression may result in substantial neurological improvement, although residual tissue may regrow. This case further demonstrates the value of follow-up imaging and repeated surgical intervention when complete excision cannot be achieved.

## Data Availability

The original contributions presented in the study are included in the article/supplementary material, further inquiries can be directed to the corresponding authors.
